# K63 ubiquitin chains target NLRP3 inflammasome for autophagic degradation in ox-LDL-stimulated THP-1 macrophages

**DOI:** 10.18632/aging.102710

**Published:** 2020-01-29

**Authors:** Zhenfeng Zhou, Xiaoyan Zhu, Ruihua Yin, Tianwei Liu, Shaonan Yang, Lingyan Zhou, Xudong Pan, Aijun Ma

**Affiliations:** 1 Department of Neurology, the Affiliated Hospital of Qingdao University, Qingdao 266000, Shandong, China; 2 Department of Intensive Care Unit, the Affiliated Hospital of Qingdao University, Qingdao 266000, Shandong, China; 3 Institute of Cerebrovascular Diseases, the Affiliated Hospital of Qingdao University, Qingdao 266000, Shandong, China

**Keywords:** atherosclerosis, NLRP3 inflammasome, autophagy, p62, K63 polyubiquitination

## Abstract

Inflammation, especially involving the NLRP3 inflammasome, is critical to atherosclerotic plaque formation. Enhanced autophagy can inhibit the development of atherosclerosis, and recent studies have revealed that NLRP3 inflammasome can be degraded by autophagy in atherosclerosis. In the present study, we established a foam-cell model to investigate the impact of oxidized low density lipoproteins (ox-LDLs) on autophagy and the inflammasome in atherosclerosis-related inflammation. We observed that ox-LDLs activated NLRP3 inflammasomes in macrophages and restricted autophagy in a time-and dose-dependent manner. We further observed through immunoprecipitation and siRNA knockdown that autophagic degradation of the NLRP3 inflammasome is dependent on K63 polyubiquitation of its NLRP3 subunit and subsequent binding by the adaptor protein p62. Our findings uncover a mechanism by which autophagy inhibits inflammation in atherosclerosis and the role of K63 in that process.

## INTRODUCTION

Ischemic stroke is a debilitating and potentially lethal disease, and the leading cause of death in modern society. The main pathogenesis of ischemic stroke is considered to be atherosclerosis [1], a proven chronic inflammatory disease [2]. High levels of interleukin-1β (IL-1β), found in atherosclerotic artery specimens [3], accelerate the progression of atherosclerosis by activating endothelial cells and macrophages, which promote the generation of adhesion molecules that recruit leukocytes [4, 5] and stimulate smooth muscle cell proliferation [6]. Recent studies have demonstrated that increased IL-1β in atherosclerotic plaque is primarily the result of activation of NLRP3 inflammasomes [7, 8]. Thus, exploring the inflammatory mechanism of atherosclerosis and manipulating the function of NLRP3 inflammasomes have emerged as new approaches to understanding and treating the disease.

The NLRP3 inflammasome consists of three units: a sensor, nucleotide-binding domain and leucine-rich repeatprotein-3 (NLRP3); an adaptor, apoptosis-associated speck-like protein containing a caspase recruitment domain (ASC); and an effector, protease caspase-1 [9]. When ligands bind to the leucine-rich repeat domain of NLRP3, NLRP3, ASC, and pro-caspase-1 oligomerize to form inflammasomes and convert dormant pro-caspase-1 into active capase-1. This, in turn, processes pro-IL-1β into mature IL-1β [10, 11].

Autophagy is a highly conserved cellular process through which misfolded proteins and excessive signaling molecules are degraded by a structure known as the autophagosome, playing a crucial role in intracellular homeostasis and adaptation to environmental changes [12–14]. Autophagic adaptor protein p62 (SQSTM1) binds and targets the substrates of autophagosomes for degradation [15, 16]. However, autophagy itself is a non-specific system, and the recognition of its substrates by p62 requires the assistance of the ubiquitination system, which identifies and labels target proteins with specific ubiquitin chains, mainly lysine 63(K63) and lysine 48(K48) polyubiquitin chains [17, 18]. P62 discerns these polyubiquitin chains to identify its targets [19].

Recent studies have shown that autophagy can degrade inflammasomes to play an anti-inflammatory role, and the process involves ubiquitination of inflammasome constituent proteins. In a dsDNA-induced acute inflammatory cell model, autophagy identified K63 ubiquitinated ASC to degrade AIM2 inflammasomes (reactive inflammasomes of dsDNA) [20]. Dopamine promotes the autophagic degradation of NLRP3 inflammasomes by enhancing the K48-linked ubiquitination of NLRP3, thereby playing a protective role against Parkinson's disease, neuritis, and peritonitis [21]. NLRP3 inflammasomes are also autophagocytosed in atherosclerosis [22].

In the present study, a foam-cell model was established to investigate the impact of oxidized low density lipoproteins (ox-LDLs) on autophagy and inflammasomes, and the effect of autophagy on inflammation in atherosclerosis. Furthermore, we investigated the specific ubiquitin chain and molecular mechanism through which autophagy recognizes the NLRP3 inflammasome.

## RESULTS

### Ox-LDLs activate NLRP3 inflammasome in a time- and dose-dependent manner

**Figure 1:**
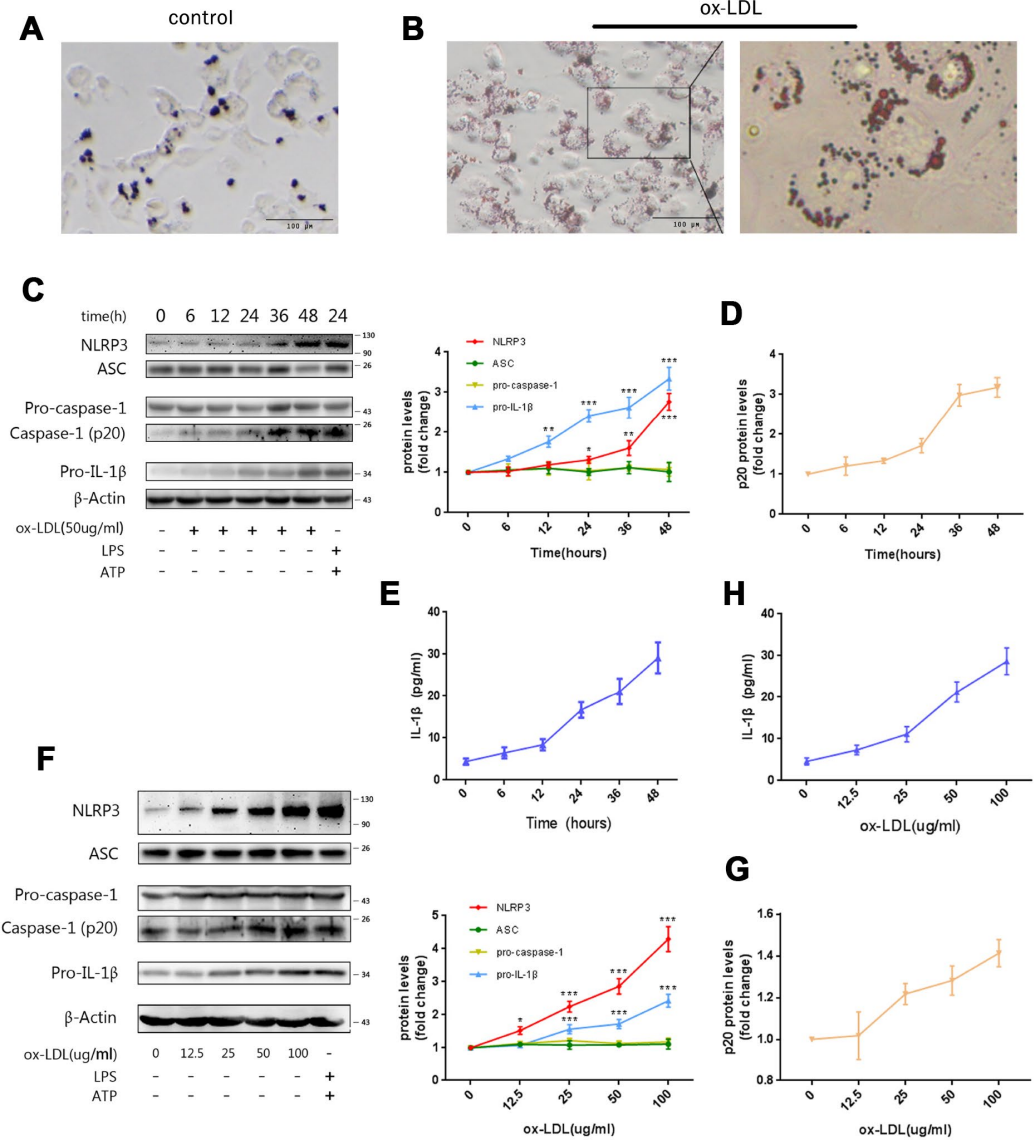
**Ox-LDLs activate NLRP3 inflammasomes in a time- and dose-dependent manner.** (**A** and **B**) Representative Oil Red O staining images of Mφ treated with (**B**) or without (**A**) ox-LDLs. The right image in (**B**), 4× enlargement of the outlined area at the left. Scale bars, 100 um. (**C**) The immunoblot analysis of lysates of Mφ treated with ox-LDLs (50 ug/ml) for a series of time intervals. (**C** and **D**) The densitometric analysis of the NLRP3, ASC, pro-caspase-1, pro-IL-1β (**C**) and p20 (**D**) signal *vs.* time, which was normalized to β-actin. (**E**) The ELISA of IL-1β in supernatants obtained from (**C**). (**F**) The immunoblot analysis of lysates of Mφ treated with various doses of ox-LDLs for 24 hours. (**F** and **G**) The densitometric analysis of the NLRP3, ASC, pro-caspase-1, pro-IL-1β (**F**) and p20 (**G**) signal *vs.* ox-LDL concentrations, which were normalized to β-actin. (**H**) The ELISA of IL-1β in supernatants obtained from (**F**). The data are presented as mean ± SD (*n*=3); * denotes statistical significance by one-way analysis of variance (ANOVA) with *post hoc* Dunnett’s multiple comparisons test when compared to 0 hour or 0 ug/ml ox-LDLs. *P<0.05, **P<0.01, ***P<0.001.

In order to establish the foam-cell models, THP-1 derived macrophages (Mφ) were stimulated with ox-LDLs for 24 hours, and stained with Oil Red O. As presented in Figure 1A and 1B**,** obvious lipid droplets were observed in ox-LDL-challenged Mφ. In sharp contrast, no lipid droplet formation was observed in the control group.

In order to determine whether ox-LDLs activate NLRP3 inflammasomes, Mφ were treated with ox-LDLs for a series of time intervals (0-48 hours) and concentrations (0-100 ug/ml). Then, the expression of NLRP3, ASC, pro-caspase-1, pro-IL-1β, and activated caspase-1 (p20) in cell lysates was measured by western blot, while the expression of IL-1β in the supernatants was determined by ELISA. We found that the expression of NLRP3 and pro-IL-1β, and the production of activated caspase-1 (p20) (Figure 1C, 1D) and IL-1β (Figure 1E) increased with the prolongation of stimulation time. Furthermore, the concentration gradient ox-LDL stimulation experiment revealed that the expression of NLRP3, pro-IL-1β, and p20 (Figure 1F, 1G), and the level of supernatant IL-1β (Figure 1H) were increased by ox-LDLs in a dose-dependent manner. Remarkably, the expression of ASC and pro-caspase-1 did not change in either experiment (Figure 1C and 1F).

These data indicate that ox-LDLs can activate NLRP3 inflammasomes, possibly by promoting the expression of NLRP3 and pro-IL-1β. Consistent with the findings in LPS-induced inflammation models, the expression of ASC and pro-caspase-1 were not affected in the process [23]. Overall, these data suggest that ox-LDLs can activate the NLRP3 inflammasome in a time- and dose-dependent manner.

### ox-LDLs restrict autophagy in a time- and dose-dependent manner

**Figure 2:**
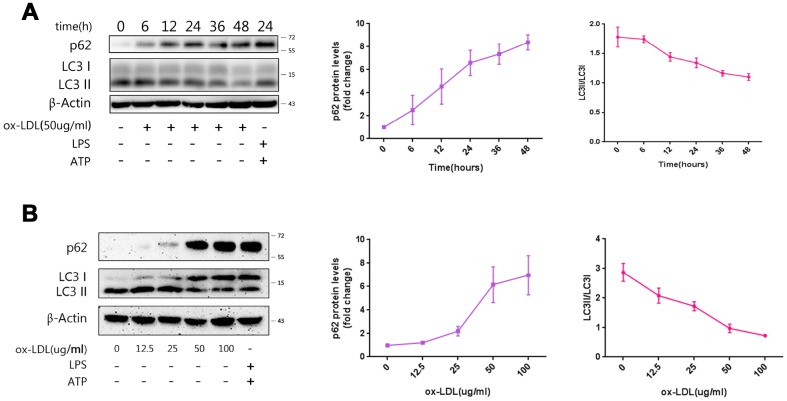
**Ox-LDLs inhibit autophagy in a time- and dose-dependent manner.** (**A**) The immunoblot analysis of lysates of Mφ treated with ox-LDLs (50 ug/ml) for a series of time intervals. (**B**) The immunoblot analysis of lysates of Mφ treated with various doses of ox-LDLs for 24 hours. (**A** and **B**) The densitometric analysis of the p62 signal and LC3II/LC3I ratio *vs.* time (**A**) and ox-LDL concentrations (**B**), which was normalized to β-actin. The data are presented as mean ± SD (*n*=3).

Next, the influence of ox-LDLs on autophagy was investigated. LC3II participates in the formation of autophagosomes, and increased LC3II/LCI ratio represents enhancement of autophagy. On the contrary, elevation of p62 indicates reduced autophagy [15]. The lysates from cells exposed to ox-LDLs in the previous experiment were subjected to western blot. As demonstrated in Figure 2A, the expression of p62 increased, and the LC3II/LCI ratio decreased with ox-LDL exposure in a time-dependent manner. Furthermore, the concentration gradient ox-LDL stimulation experiments revealed that the expression of p62 increased and the LC3II/LCI ratio decreased in a dose-dependent manner (Figure 2B). Overall, these results suggest that ox-LDLs suppress autophagy in a time- and dose-dependent manner.

### Autophagy inhibits ox-LDL-induced NLRP3 inflammasome activation by degrading NLRP3 and ASC

**Figure 3:**
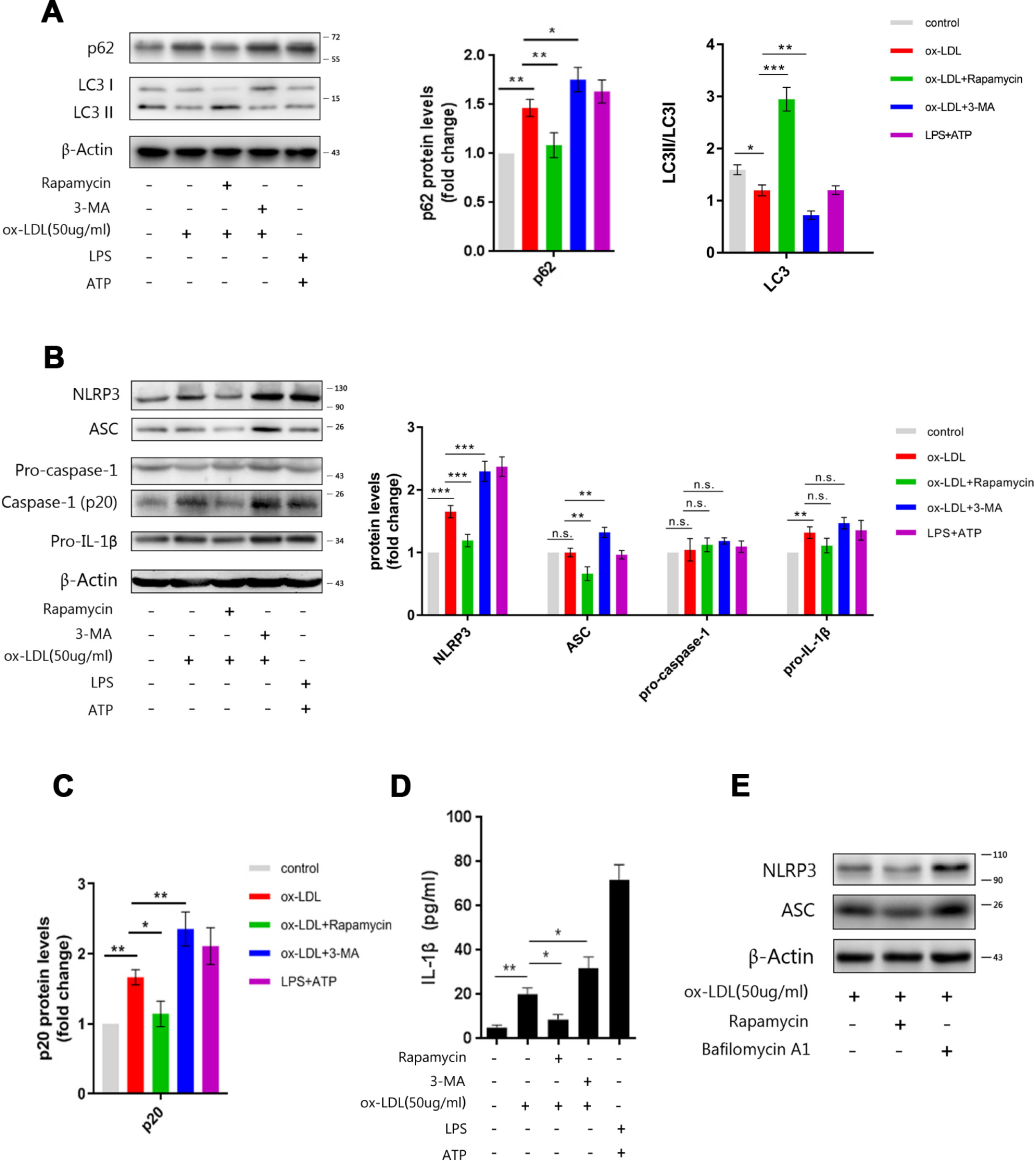
**Manipulating the autophagy affects NLRP3 inflammasomes.** (**A**) The immunoblot analysis of lysates of Mφ, which were left untreated or treated with rapamycin or 3-MA, and subsequently stimulated with ox-LDLs (50 ug/ml), or LPS and ATP for 24 hours. (**B**) The immunoblot analysis of lysates of Mφ, which were left untreated or treated with rapamycin or 3-MA, and subsequently stimulated with ox-LDLs (50 ug/ml), or LPS and ATP for 24 hours. (**A**–**C**) The densitometric analysis of the p62 signal and LC3II/LC3I ratio (**A**), the NLRP3, ASC, pro-caspase-1 and pro-IL-1β signal (**B**), and the p20 signal (**C**), which were normalized to β-actin. (**D**) The ELISA of IL-1β in the supernatants obtained from (**B**). (**E**) The immunoblot analysis of lysates of Mφ, which were left untreated or treated with rapamycin or bafilomycin A1, and subsequently stimulated with ox-LDLs (50 ug/ml) for 24 hours. The data are presented as mean ± SD (*n*=3); * denotes the statistical significance by one-way ANOVA with *post hoc* Dunnett’s multiple comparisons test. *P<0.05, **P<0.01, ***P<0.001.

Rapamycin, an autophagy enhancer, and 3-MA, an inhibitor, [24] were administered to Mφ at one hour prior to ox-LDL stimulation. The western blot revealed that treatment with rapamycin decreased p62 in the cell lysates, but increased the LC3II/LCI ratio; conversely, treatment with 3-MA increased p62, but decreased the LC3II/LCI ratio (Figure 3A).

Next, we measured activated caspase-1 and mature IL-1β in Mφ, which had been treated with rapamycin or 3-MA and subsequently stimulated with ox-LDLs. Evidently, restricting autophagy with 3-MA resulted in more inflammasome activity, as reflected by the increase in p20 and IL-1β in cell lysates and the supernatant. Furthermore, the elevation of autophagy with rapamycin resulted in less inflammasome activity,.as reflected by lesser p20 and IL-1β (Figure 3B–3D). These data indicate that autophagy can regulate ox-LDL-induced NLRP3 inflammasome activation.

In order to further investigate the mechanism by which autophagy affects inflammasome activation, we measured the expression of NLRP3, ASC, pro-caspase-1 and pro-IL-1β by western blot. As shown in Figure 3B, enhancing autophagy with rapamycin resulted in less NLRP3 and ASC, while blocking autophagy with 3-MA led to more NLRP3 and ASC in the cell lysates. However, the manipulation of autophagy did not cause significant changes in the expression of pro-caspase-1 and pro-IL-1β. These data show that NLRP3 and ASC are regulated by autophagy. Next, bafilomycin A1, an autophagy inhibitor that blocks autophagosome-lysosome fusion, was administered to foam-cells to clarify whether NLRP3 and ASC were actually degraded by autophagy. As shown in Figure 3E, rapamycin dampened the expression of NLRP3 and ASC, while bafilomycin A1 elevated the expression of NLRP3 and ASC. This indicates that NLRP3 and ASC can be degraded *via* the autophagy process. In summary, these results suggest that autophagy may inhibit the activation of NLRP3 inflammasomes by degrading NLRP3 and ASC, but not pro-caspase-1 and pro-IL-1β.

### P62 is essential for autophagic degradation of NLRP3 inflammasomes

**Figure 4:**
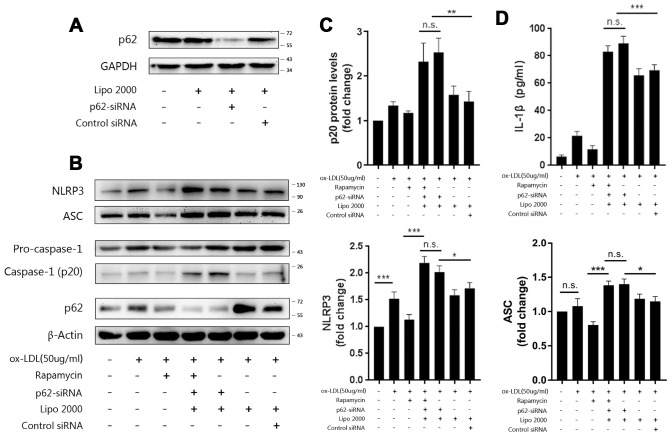
**P62 mediates the autophagic regulation of NLRP3 inflammasomes.** (**A**) The expression of p62 in the lysates of Mφ, which were left untransfected or transfected with vehicle, p62-siRNA, or control siRNA for 48 hours. (**B**) The immunoblot analysis of the lysates of Mφ, which were left untransfected or transfected with vehicle, p62-siRNA, or control siRNA for 24 hours, and subsequently treated with or without rapamycin, and stimulated with ox-LDLs (50 ug/ml) for another 24 hours. Rapamycin was administered to cells for one hour prior to ox-LDL stimulation. (**B** and **C**) The densitometric analysis of the NLRP3, ASC, (**B**) and p20 (**C**) signal, which were normalized to β-actin. (**D**) The ELISA of IL-1β in the supernatants obtained from (**B**). The data are presented as mean ± SD (*n*=3); * denotes the statistical significance by one-way ANOVA with *post hoc* Dunnett’s multiple comparisons test. *P<0.05, **P<0.01, ***P<0.001.

P62 is an important adaptor protein in autophagy, which identifies, binds, and targets substrates to the autophagosome for degradation [16]. In order to verify whether p62 mediates the recognition of NLRP3 inflammasomes by autophagy, p62-siRNA was transfected into Mφ. Cell lysates from Mφ transfected with p62-siRNA contained less p62 (Figure 4A) than controls. The ablation of p62 with siRNA in foam-cells led to more NLRP3, ASC (Figure 4B), and p20 (Figure 4B and 4C) in the cell lysates and IL-1β (Figure 4D)in the supernatants, when compared to the control siRNA group; this was similar to the effects of 3-MA and bafilomycin A1.

In order to further evaluate the role of p62 in autophagy and inflammation, rapamycin was administered to Mφ transfected with p62-siRNA at one hour prior to ox-LDL stimulation. As shown in Figure 4B–4D, the expression of NLRP3, ASC and p20 in the cell lysates, and the level of IL-1β in the supernatants were not depressed by rapamycin, indicating that the effect of rapamycin in limiting inflammation by triggering autophagy was blocked. Overall, these data suggest that p62 plays an important role in the foam-cell model, and mediates the regulation of NLRP3 inflammasome by autophagy.

### NLRP3 is the main target of p62

**Figure 5:**
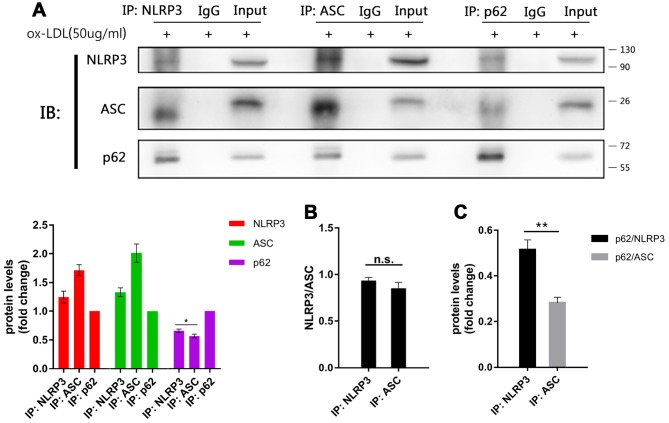
**The main target of p62 is more likely to be NLRP3.** (**A** above) The immunoblot analysis of the NLRP3, ASC and p62 immunoprecipitates of cell lysates obtained from Mφ, which had been stimulated with ox-LDLs (50 ug/ml) for 24 hours. (**A** below) The densitometric analysis of the NLRP3, ASC and p62 signal, which were normalized to the corresponding signals in the p62 immunoprecipitates. (**B**) The densitometric analysis of the ratios of NLRP3/ASC obtained from the NLRP3 immunoprecipitates and the ASC immunoprecipitates. (**C**) The densitometric analysis of p62 signal detected in the NLRP3 immunoprecipitates and the ASC immunoprecipitates, and normalized to the corresponding NLRP3 and ASC signal respectively. The data are presented as mean ± SD (*n*=3); * denotes the statistical significance by one-way ANOVA with *post hoc* Dunnett’s multiple comparisons test (**A**) or by t-test (**B** and **C**). *P<0.05, **P<0.01, ***P<0.001.

According to the present results, the expression of NLRP3 and ASC, rather than pro-caspase-1 and pro-IL-1β, significantly vary with the manipulation of autophagy, which implies that NLRP3 and ASC are targets of autophagy. Previous studies have shown that both NLRP3 and ASC can interact and co-localize with p62 alone [20, 25]. So, immunoprecipitation was performed to clarify whether NLRP3 or ASC is the major target of autophagy, or whether they play equal roles in mediating the autophagic degradation of NLRP3 inflammasomes. As shown in Figure 5, both NLRP3 and ASC were immunoprecipitated from cell lysates with the p62 antibody (Figure 5A). When the cell lysates were immunoprecipitated with NLRP3 or ASC antibodies, the ratios of NLRP3 to ASC obtained from the NLRP3 and the ASC immunoprecipitates were the same (Figure 5A and 5B). These results indicate that there is a stable combination between NLRP3 and ASC and rules out the influence of the disassembly of NLRP3 and ASC on p62. However, more p62 was detected in the NLRP3 immunoprecipitates than in the ASC immunoprecipitates (Figure 5A). This trend was more significant when the signals of p62 obtained from the NLRP3 immunoprecipitates or the ASC immunoprecipitates were normalized to NLRP3 or ASC, respectively (Figure 5C). These results indicate that more p62 detached from the NLRP3 and ASC complex during ASC immunoprecipitation and the combination of p62 to the NLRP3 and ASC complex is preserved better when immunoprecipitated with NLRP3 antibodies. These results suggest that NLRP3 may be the major or direct target of p62.

### P62 binds to NLRP3 *via* the K63 polyubiquitin chains

**Figure 6:**
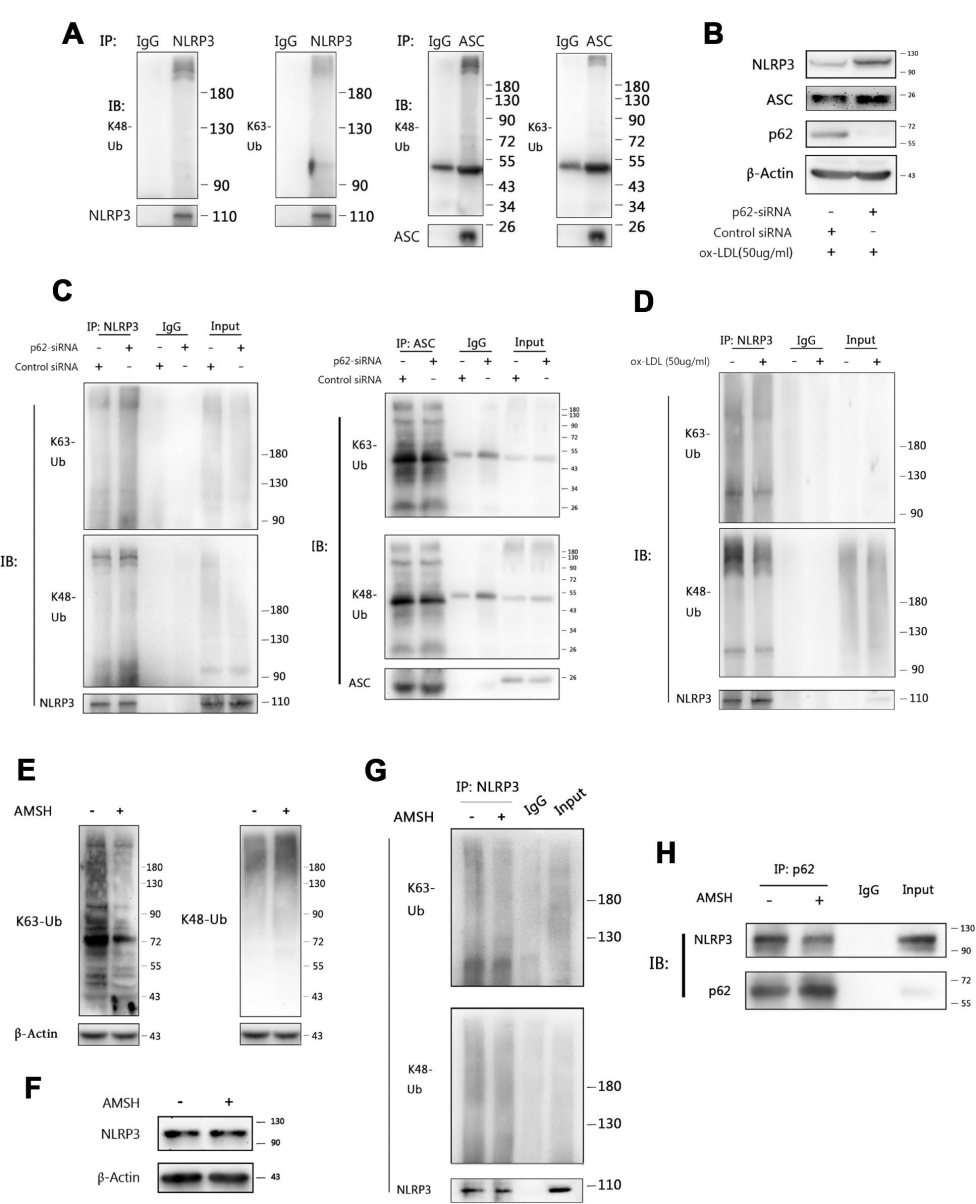
**P62 binds to NLRP3 *via* the K63 polyubiquitin chains.** (**A**) The immunoblot analysis of NLRP3 and ASC immunoprecipitates of Mφ stimulated with ox-LDLs (50 ug/ml) for 24 hours. (**B**) The immunoblot analysis of the total lysates of Mφ transfected with control siRNA or p62-siRNA for 24 hours, and subsequently stimulated with ox-LDLs (50 ug/ml) for another 24 hours. (**C**) The immunoblot analysis of NLRP3 (left) and ASC (right) immunoprecipitates of Mφ treated as described in (**B**). (**D**) The immunoblot analysis of NLRP3 immunoprecipitates of Mφ treated with or without ox-LDL (50 ug/ml) for 24 hours. (**E**) The detection for K63 (left) and K48 (right) polyubiquitin chains in the total foam-cell lysates pretreated with or without AMSH by immunoblotting. Prior to immunoblotting, 50 ul of ox-LDL stimulated Mφ lysates were administered with or without 1 ul of AMSH (AMSH concentration: 500 nM), and incubated at 37°C for 30 minutes. (**F**) The immunoblot analysis of NLRP3 and β-Actin in the total foam-cell lysates pretreated with or without AMSH (500 nM), as described in (**E**). (**G**) The NLRP3 immunoprecipitates obtained from ox-LDL stimulated Mφ lysates were treated with or without AMSH (500 nM), as described in (**E**), and subsequently subjected to immunoblotting. (**H**) The p62 immunoprecipitates obtained from foam-cell lysates were treated with or without AMSH (500 nM), as described in (**E**), and subsequently subjected to immunoblotting. All experiments were independently repeated for at least three times.

We next sought to determine how p62 recognizes NLRP3 inflammasomes. P62 contains an ubiquitin binding domain (UBA). Hence, immunoprecipitation was performed to determine whether NLRP3 or ASC was ubiquitinated in ox-LDL-stimulated Mφ. As shown in Figure 6A, both lysine 48 (K48)- and lysine 63 (K63)-linked polyubiquitin chains were detected in the NLRP3 and ASC immunoprecipitates obtained from foam-cell lysates. Ablating p62 with p62-siRNA increased the NLRP3 and ASC expression (Figure 6B). Further investigation revealed that the K63, rather than the K48, polyubiquitin chains dramatically accumulated on NLRP3 when p62-siRNA was transfected into Mφ. Furthermore, the K48 and K63 polyubiquitin chains that attached to ASC did not significantly change, when compared with the control siRNA groups (Figure 6C). Interestingly, it was found that ox-LDL stimulation slightly reduced the K48 and K63 ubiquitin chains attached to NLRP3 (Figure 6D). This is consistent with the discovery that NLRP3 undergoes de-ubiquitination during NLRP3 inflammasome activation [26]. These data suggest that the accumulation of K63 polyubiquitin chains on NLRP3 is a specific result of the p62 ablation. These results indicate that K63 polyubiquitin chains play an important role in the binding of p62 to NLRP3, and further confirm that NLRP3 is the main target of p62 in the autophagic regulation process of inflammation.

In order to further certify these findings, AMSH (an associated molecule with an Src homology 3 domain of the signal transducing adaptor molecule), which is a K63-linked polyubiquitin chain-specific deubiquitinating enzyme [27], was used to trim the K63 polyubiquitin chains on NLRP3. First, immunoblotting was performed, and it was found that AMSH hydrolyzed the K63 polyubiquitin chains without affecting the K48 polyubiquitin chains (Figure 6E) or NLRP3 (Figure 6F). Next, the NLRP3 immunoprecipitates were treated with AMSH. Consistent with the previous results, AMSH ablated the K63 polyubiquitin chains in the precipitates, while the K48 polyubiquitin chains were exempted (Figure 6G). Finally, the p62 immunoprecipitates were pretreated with AMSH, and less NLRP3 was detected by western blot than in the control group (Figure 6H). Overall, these results indicate that p62 mediates the regulation of NLRP3 inflammasomes through autophagy by recognizing the K63 polyubiquitin chains on NLRP3.

## DISCUSSION

The present study uncovered the molecular mechanism and role of ubiquitination in mediating the identification of NLRP3 inflammasomes by autophagy. In the foam- cell models, ox-LDLs on their own activated NLRP3 inflammasomes and restricted autophagy. Meanwhile, regulation of NLRP3 inflammasomes is mediated by the autophagy adaptor protein p62 and that this process was achieved through the recognition of p62 to the K63 ubiquitin chains on the NLRP3 protein.

The effect of ox-LDLs on NLRP3 inflammasomes has long been under discussion. One possible mechanism by which ox-LDLs activate NLRP3 inflammasomes is that ox-LDLs promote the production of cholesterol crystals, which in turn induce the expression and activation of NLRP3 inflammasomes [7]. However, ox-LDLs also inhibit LPS-induced inflammation by blocking the binding of NF-κB to the DNA in a dose-dependent manner [28]. Yury I. Miller hypothesized that low level and transient ox-LDL stimulation dampens the inflammatory response. However, under conditions of hyperlipidemia, the increased and prolonged production of ox-CE and ox-PE (oxidized cholesteryl esters and oxidized phosphatidylethanolamine, the components of ox-LDLs) may result in chronic inflammation [29]. It was demonstrated in the present study that ox-LDLs activate NLRP3 inflammasomes in a time-and dose-dependent manner, and long-term ox-LDL stimulation activates NLRP3 inflammasomes, even when the stimulating concentration is lower than 10ug/ml.

The finding that inflammasome itself can be regulated by autophagy is a new discovery in recent years [14]. In dsDNA stimulated macrophages, autophagy has been reported to be able to degrade ASC to block the assembly of AIM2 inflammasomes [20]. Autophagy has also been proven to engulf NLRP3 to play an anti-inflammatory role in Parkinson’s disease [21]. Furthermore, we found in ox-LDL-induced foam-cells that autophagy regulates inflammation by degrading the NLRP3 inflammasome subunit—NLRP3 and ASC. Further investigation revealed that the main target of autophagy is more likely to be NLRP3, because the combination of p62 to the NLRP3 and ASC complex is preserved better when immunoprecipitated with NLRP3 antibodies and that the ubiquitination modification on ASC remains unchanged after knocking down the expression of p62.

The role of p62 is crucial for autophagy to identify substrates. Mutations in the ubiquitin associated domain (UBA) deprive p62 of the ability to recognize K48 polyubiquitin chains, resulting in Paget’s disease [30]. Autophagy moderates atherosclerosis by engulfing misfolded proteins bounded by p62 [31]. Recent studies have revealed that p62 efficiently targets proteins labeled by two or more ubiquitin chains composed of at least three ubiquitin moieties, and crosses link with each other to cluster substrates [32]. This is the result of the viscous liquid-like properties of p62, which promote a phase separation after p62 covalently combines with the ubiquitin chains attached on the substrates. These cargos further recruit LC3 and drive the assembly of autophagosome around themselves [33]. In agreement with these findings, we found that autophagy regulates NLRP3 inflammasomes in atherosclerosis through the binding of p62 to NLRP3, since depleting p62 blocks the NLRP3 inflammasome regulating effect of autophagy, and reversing the anti-inflammatory role of rapamycin.

Ubiquitination has been proven to participate in the activation and degradation of inflammasomes. G5, which is a small molecule inhibitor of deubiquitination, critically regulates inflammasome activity by dampening the deubiquitination of NLRP3 [26]. The K63 polyubiquitin modification of pro-IL-1β has been considered as a prerequisite for the binding of activated caspase-1 to pro-IL-1β [27]. Furthermore, the K48 ubiquitination of NLRP3 promotes its proteasomal degradation [34]. It appears that the function of ubiquitination varies with the ubiquitin chain types and the proteins targeted. In the present study, it was revealed that ablating p62 results in increase in K63 polyubiquitin chains attached to NLRP3, and the disassembly of K63 polyubiquitin chains on NLRP3 with AMSH, leading to decreased NLRP3 binding with p62. These results suggest that the autophagic adaptor protein p62 detects NLRP3 by identifying the accumulated K63 polyubiquitin chains on it. The slightly longer K48 polyubiquitin chains on NLRP3, when ablating p62 with p62-siRNA, may be the result of the compensatory increase of proteasome degradation.

In summary, we demonstrated that in ox-LDL-stimulated foam-cells, ox-LDLs activate NLRP3 inflammasomes while inhibiting autophagy, which exacerbates the inflammatory response in foam-cells. Manipulating autophagy could be an effective approach to regulate the function of NLRP3 inflammasomes. It is noteworthy that the possible mechanism is that the autophagy engulfs NLRP3 *via* the recognition by p62 of the K63 ubiquitin chains on NLRP3. This NLRP3 inflammasome-regulating mechanism may help lead to development of specific activators or inhibitors, and fruitful atheroprotective measures in the future.

## MATERIALS AND METHODS

### Reagents and antibodies

ox-LDLs were purchased from Yiyuanbiotech (Guangzhou, China). Phorbol-12-myristate-13-acetate (PMA), rapamycin, 3-methyladenine (3-MA), bafilomycin A1 and protease inhibitor cocktails were purchased from MCE (Monmouth Junction, USA). LPS and ATP were purchased from Sigma (St Louis, MO, USA). Lipofectamine™ 2000 was purchased from Invitrogen (Carlsbad, USA). Antibody to GAPDH, antibody to β-actin and HRP-conjugated secondary polyclonal antibodies to mouse and rabbit IgGs were purchased from Elabscience (Wuhan, China). Rabbit monoclonal anti-NLRP3, anti-ASC, anti-SQSTM1/p62, anti-LC3B, antibody to K63-linked polyubiquitin chains, antibody to K48-linked polyubiquitin chains and p62-siRNA, and control siRNA were purchased from Cell Signaling Technology (Boston, USA). Rabbit monoclonal anti-IL-1β, anti-caspase-1 were purchased from Abcam (Cambridge, MA, USA). For the immunoprecipitation, polyclonal rabbit anti-SQSTM1/p62 was purchased from Abcam (Cambridge, MA, USA). Monoclonal mouse anti-SQSTM1/p62, anti-NLRP3, anti-ASC and protein A/G Plus-agarose were purchased from Santa Cruz Biotechnology (Santa Cruz, CA, USA). AMSH (the JAMM/MPN+ family of K63-specific DUB) was purchased from Boston Biochem (Cambridge, MA, USA).

### Cell culture

Human THP-1 monocytes purchased from Shanghai Institutes for Biological Sciences (SIBS, Shanghai, China) were routinely cultured in 1640 medium supplemented with 10% fetal bovine serum (FBS; Biological Industries, Israel) and 1% penicillin-streptomycin (HyClone, USA) at 37°C with 5% CO_2_. Prior to the experiments, THP-1 cells (4×10^5^ cells/ml) were differentiated into macrophages with 25 ng/ml of PMA for 24 hours, and incubated in fresh culture medium for another 24 hours. These cells were referred to as THP-1 Mφ. Mφ were treated with 50 ug/ml of ox-LDLs for 24 hours, or according to the concentration and time indicated in the specific experiment to establish the foam-cells model. In autophagy modulation experiments, 25 nM of rapamycin, 5 mM of 3-MA or 15 nM bafilomycin A1 were administered to the Mφ at one hour prior to ox-LDLs and incubated for 24 hours. LPS and ATP were used at concentration of 100 ng/ml and 5 mM, respectively.

### Oil red O staining

Next, 2×10^5^ cells/ml of THP-1 monocytes were seeded into 6-well plates and sequentially treated with PMA and ox-LDLs, as described. After 72 hours, these cells were rinsed with PBS twice, and fixed with 4% paraformaldehyde for 25 minutes. Then, these cells were rinsed with 60% isopropanol for 15 seconds, stained with Oil Red O working solution for 30 minutes at 37°C, and rinsed with PBS twice and 60% isopropanol for 10 seconds, successively. Afterwards, these cells were immediately rinsed with PBS again, and photographed under a microscope.

### Transfection with siRNA

THP-1 cells were seeded at a density of 5×10^5^ cells/ml, differentiated with PMA, as described, and incubated in antibiotic-free medium for 24 hours. Lipofectamine™ 2000 was used according to manufacturer's instructions to transfect the siRNA for six hours. Then, these cells were cultured in antibiotic-free medium for 18 hours prior to subsequent experiments.

### Enzyme-linked immunosorbent assay (ELISA)

The IL-1β level in the cell culture supernatant was determined using IL-1β ELISA Kits (Elabscience, Wuhan, China), and performed according to manufacturer’s instructions.

### Western blot

Cells were lysed on ice with standard RIPA buffer containing PMSF and a protease inhibitor cocktail for 30 minutes. After centrifugation for 10 minutes at 4°C, the supernatants were collected and subjected to 6-12% SDS-polyacrylamide gel electrophoresis. Then, the protein samples on the gel were transferred onto polyvinylidene difluoride membranes (Millipore Co, NJ, USA). After blocking in 5% skim milk at room temperature for 1.5 hours, these membranes were incubated with the appropriate primary antibody overnight at 4°C. The next day, the membranes were washed with TBST thrice, and incubated with HRP-conjugated anti-rabbit or mouse IgG antibodies for 1.5 hours at room temperature. Then, the bands were developed using Pierce ECL (Millipore Co, NJ, USA), and the images were gathered and analyzed using the NIH Image J software.

### Immunoprecipitation

4×10^6^ cells in 10-cm dishes were lysed on ice in lysis buffer (20 mM of Tris [pH 7.5], 150 mM of NaCl, and 1% Triton X-100) with PMSF and the protease inhibitor cocktail, and sonicated. Then, the lysates were incubated with the appropriate antibodies for 2-10 hours at 4°C prior to the addition of protein A/G Plus-agarose. Afterwards, the immunoprecipitates were collected and washed four times with the lysis buffer, and subjected to immunoblotting.

### DUB assays

Cells in a 10-cm dish were lysed and immunoprecipitated with anti-p62, as previously described. The precipitates were washed three times with lysis buffer, and an additional two times with a reaction buffer (50 mM of Tris, pH 7.5, 150 mM of sodium chloride, 25 mM of potassium chloride, 5 mM of magnesium chloride and 1 mM of DTT). Then, the precipitates were collected and resuspended in 50 ul of reaction buffer, containing 1 ul of AMSH. After incubation at 37°C for 30 minutes, the precipitates were subjected to two washes, and heated in 2× loadingbuffer.

### Statistical analysis

Data are presented as mean ± standard deviation (SD). All cell experiments were independently performed at least three times. All statistical analyses were performed using Graph Pad Prism 7 software (GraphPad Software, San Diego, CA, USA). *P*<0.05 were considered statistically significant.

## References

[1] Braunwald E. Shattuck lecture—cardiovascular medicine at the turn of the millennium: triumphs, concerns, and opportunities. N Engl J Med. 1997; 337:1360–69. 10.1056/NEJM199711063371906 9358131

[2] Libby P. Inflammation in atherosclerosis. Arterioscler Thromb Vasc Biol. 2012; 32:2045–51. 10.1161/ATVBAHA.108.179705 22895665 PMC3422754

[3] Galea J, Armstrong J, Gadsdon P, Holden H, Francis SE, Holt CM. Interleukin-1 beta in coronary arteries of patients with ischemic heart disease. Arterioscler Thromb Vasc Biol. 1996; 16:1000–06. 10.1161/01.ATV.16.8.1000 8696938

[4] Fearon WF, Fearon DT. Inflammation and cardiovascular disease: role of the interleukin-1 receptor antagonist. Circulation. 2008; 117:2577–79. 10.1161/CIRCULATIONAHA.108.772491 18490534

[5] Abbate A, Van Tassell BW, Biondi-Zoccai GG. Blocking interleukin-1 as a novel therapeutic strategy for secondary prevention of cardiovascular events. BioDrugs. 2012; 26:217–33. 10.1007/BF03261881 22571369

[6] Libby P, Warner SJ, Friedman GB. Interleukin 1: a mitogen for human vascular smooth muscle cells that induces the release of growth-inhibitory prostanoids. J Clin Invest. 1988; 81:487–98. 10.1172/JCI113346 3276731 PMC329596

[7] Duewell P, Kono H, Rayner KJ, Sirois CM, Vladimer G, Bauernfeind FG, Abela GS, Franchi L, Nuñez G, Schnurr M, Espevik T, Lien E, Fitzgerald KA, et al. NLRP3 inflammasomes are required for atherogenesis and activated by cholesterol crystals. Nature. 2010; 464:1357–61. 10.1038/nature08938 20428172 PMC2946640

[8] Zhuang T, Liu J, Chen X, Zhang L, Pi J, Sun H, Li L, Bauer R, Wang H, Yu Z, Zhang Q, Tomlinson B, Chan P, et al. Endothelial Foxp1 Suppresses Atherosclerosis via Modulation of Nlrp3 Inflammasome Activation. Circ Res. 2019; 125:590–605. 10.1161/CIRCRESAHA.118.314402 31318658

[9] Schroder K, Tschopp J. The inflammasomes. Cell. 2010; 140:821–32. 10.1016/j.cell.2010.01.040 20303873

[10] Martinon F, Burns K, Tschopp J. The inflammasome: a molecular platform triggering activation of inflammatory caspases and processing of proIL-beta. Mol Cell. 2002; 10:417–26. 10.1016/S1097-2765(02)00599-3 12191486

[11] Wang L, Manji GA, Grenier JM, Al-Garawi A, Merriam S, Lora JM, Geddes BJ, Briskin M, DiStefano PS, Bertin J. PYPAF7, a novel PYRIN-containing Apaf1-like protein that regulates activation of NF-kappa B and caspase-1-dependent cytokine processing. J Biol Chem. 2002; 277:29874–80. 10.1074/jbc.M203915200 12019269

[12] Deretic V, Saitoh T, Akira S. Autophagy in infection, inflammation and immunity. Nat Rev Immunol. 2013; 13:722–37. 10.1038/nri3532 24064518 PMC5340150

[13] Kim KH, Lee MS. Autophagy—a key player in cellular and body metabolism. Nat Rev Endocrinol. 2014; 10:322–37. 10.1038/nrendo.2014.35 24663220

[14] Cadwell K. Crosstalk between autophagy and inflammatory signalling pathways: balancing defence and homeostasis. Nat Rev Immunol. 2016; 16:661–75. 10.1038/nri.2016.100 27694913 PMC5343289

[15] Pankiv S, Clausen TH, Lamark T, Brech A, Bruun JA, Outzen H, Øvervatn A, Bjørkøy G, Johansen T. p62/SQSTM1 binds directly to Atg8/LC3 to facilitate degradation of ubiquitinated protein aggregates by autophagy. J Biol Chem. 2007; 282:24131–45. 10.1074/jbc.M702824200 17580304

[16] Ponpuak M, Davis AS, Roberts EA, Delgado MA, Dinkins C, Zhao Z, Virgin HW 4th, Kyei GB, Johansen T, Vergne I, Deretic V. Delivery of cytosolic components by autophagic adaptor protein p62 endows autophagosomes with unique antimicrobial properties. Immunity. 2010; 32:329–41. 10.1016/j.immuni.2010.02.009 20206555 PMC2846977

[17] Chau V, Tobias JW, Bachmair A, Marriott D, Ecker DJ, Gonda DK, Varshavsky A. A multiubiquitin chain is confined to specific lysine in a targeted short-lived protein. Science. 1989; 243:1576–83. 10.1126/science.2538923 2538923

[18] Jin L, Williamson A, Banerjee S, Philipp I, Rape M. Mechanism of ubiquitin-chain formation by the human anaphase-promoting complex. Cell. 2008; 133:653–65. 10.1016/j.cell.2008.04.012 18485873 PMC2696189

[19] Khaminets A, Behl C, Dikic I. Ubiquitin-Dependent And Independent Signals In Selective Autophagy. Trends Cell Biol. 2016; 26:6–16. 10.1016/j.tcb.2015.08.010 26437584

[20] Shi CS, Shenderov K, Huang NN, Kabat J, Abu-Asab M, Fitzgerald KA, Sher A, Kehrl JH. Activation of autophagy by inflammatory signals limits IL-1β production by targeting ubiquitinated inflammasomes for destruction. Nat Immunol. 2012; 13:255–63. 10.1038/ni.2215 22286270 PMC4116819

[21] Yan Y, Jiang W, Liu L, Wang X, Ding C, Tian Z, Zhou R. Dopamine controls systemic inflammation through inhibition of NLRP3 inflammasome. Cell. 2015; 160:62–73. 10.1016/j.cell.2014.11.047 25594175

[22] Peng S, Xu LW, Che XY, Xiao QQ, Pu J, Shao Q, He B. Atorvastatin Inhibits Inflammatory Response, Attenuates Lipid Deposition, and Improves the Stability of Vulnerable Atherosclerotic Plaques by Modulating Autophagy. Front Pharmacol. 2018; 9:438. 10.3389/fphar.2018.00438 29773990 PMC5943597

[23] He Y, Hara H, Núñez G. Mechanism and Regulation of NLRP3 Inflammasome Activation. Trends Biochem Sci. 2016; 41:1012–21. 10.1016/j.tibs.2016.09.002 27669650 PMC5123939

[24] Yang Z, Klionsky DJ. Mammalian autophagy: core molecular machinery and signaling regulation. Curr Opin Cell Biol. 2010; 22:124–31. 10.1016/j.ceb.2009.11.014 20034776 PMC2854249

[25] Mehto S, Jena KK, Nath P, Chauhan S, Kolapalli SP, Das SK, Sahoo PK, Jain A, Taylor GA, Chauhan S. The Crohn's Disease Risk Factor IRGM Limits NLRP3 Inflammasome Activation by Impeding Its Assembly and by Mediating Its Selective Autophagy. Mol Cell. 2019; 73:429–445.e7. 10.1016/j.molcel.2018.11.018 30612879 PMC6372082

[26] Py BF, Kim MS, Vakifahmetoglu-Norberg H, Yuan J. Deubiquitination of NLRP3 by BRCC3 critically regulates inflammasome activity. Mol Cell. 2013; 49:331–38. 10.1016/j.molcel.2012.11.009 23246432

[27] Duong BH, Onizawa M, Oses-Prieto JA, Advincula R, Burlingame A, Malynn BA, Ma A. A20 restricts ubiquitination of pro-interleukin-1β protein complexes and suppresses NLRP3 inflammasome activity. Immunity. 2015; 42:55–67. 10.1016/j.immuni.2014.12.031 25607459 PMC4302274

[28] Ohlsson BG, Englund MC, Karlsson AL, Knutsen E, Erixon C, Skribeck H, Liu Y, Bondjers G, Wiklund O. Oxidized low density lipoprotein inhibits lipopolysaccharide-induced binding of nuclear factor-kappaB to DNA and the subsequent expression of tumor necrosis factor-alpha and interleukin-1beta in macrophages. J Clin Invest. 1996; 98:78–89. 10.1172/JCI118780 8690807 PMC507403

[29] Miller YI, Shyy JY. Context-Dependent Role of Oxidized Lipids and Lipoproteins in Inflammation. Trends Endocrinol Metab. 2017; 28:143–52. 10.1016/j.tem.2016.11.002 27931771 PMC5253098

[30] Cavey JR, Ralston SH, Hocking LJ, Sheppard PW, Ciani B, Searle MS, Layfield R. Loss of ubiquitin-binding associated with Paget’s disease of bone p62 (SQSTM1) mutations. J Bone Miner Res. 2005; 20:619–24. 10.1359/JBMR.041205 15765181

[31] Sergin I, Evans TD, Zhang X, Bhattacharya S, Stokes CJ, Song E, Ali S, Dehestani B, Holloway KB, Micevych PS, Javaheri A, Crowley JR, Ballabio A, et al. Exploiting macrophage autophagy-lysosomal biogenesis as a therapy for atherosclerosis. Nat Commun. 2017; 8:15750. 10.1038/ncomms15750 28589926 PMC5467270

[32] Zaffagnini G, Savova A, Danieli A, Romanov J, Tremel S, Ebner M, Peterbauer T, Sztacho M, Trapannone R, Tarafder AK, Sachse C, Martens S. p62 filaments capture and present ubiquitinated cargos for autophagy. EMBO J. 2018; 37:37. 10.15252/embj.201798308 29343546 PMC5830917

[33] Sun D, Wu R, Zheng J, Li P, Yu L. Polyubiquitin chain-induced p62 phase separation drives autophagic cargo segregation. Cell Res. 2018; 28:405–15. 10.1038/s41422-018-0017-7 29507397 PMC5939046

[34] Song H, Liu B, Huai W, Yu Z, Wang W, Zhao J, Han L, Jiang G, Zhang L, Gao C, Zhao W. The E3 ubiquitin ligase TRIM31 attenuates NLRP3 inflammasome activation by promoting proteasomal degradation of NLRP3. Nat Commun. 2016; 7:13727. 10.1038/ncomms13727 27929086 PMC5155141

